# The European AntibotABE Framework Program and Its Update: Development of Innovative Botulinum Antibodies

**DOI:** 10.3390/toxins9100309

**Published:** 2017-10-02

**Authors:** Christine Rasetti-Escargueil, Arnaud Avril, Sebastian Miethe, Christelle Mazuet, Yagmur Derman, Katja Selby, Philippe Thullier, Thibaut Pelat, Remi Urbain, Alexandre Fontayne, Hannu Korkeala, Dorothea Sesardic, Michael Hust, Michel R. Popoff

**Affiliations:** 1 Institut Pasteur, Unité des Bactéries Anaérobies et Toxines, 25 Avenue du Docteur Roux, 75015 Paris, France; christelle.mazuet@pasteur.fr (C.M.); michel-robert.popoff@pasteur.fr (M.R.P.); 2 Institut de Recherche Biomédicale des Armées (IRBA-CRSSA), Département de Microbiologie, Unité de Biotechnologie des Anticorps et Des Toxins, Cedex 38702 La Tronche, France; pthullier@yahoo.com (P.T.); thibaut.pelat@biotem.fr (T.P.); 3 Institut de Recherche Biomédicale des Armées (IRBA), Département des Maladies Infectieuses, Unité Biothérapies anti-Infectieuses et Immunité, 1 Place du Général Valérie André, BP73, 91220 Brétigny-sur-Orge, France; 4 Technische Universität Braunschweig, Institut für Biochemie, Biotechnologie und Bioinformatik, Abteilung Biotechnologie, Spielmannstr. 7, 38106 Braunschweig, Germany and YUMAB GmbH, Rebenring 33, Braunschweig 38106, Germany; miethe.sem@icloud.com (S.M.); m.hust@tu-bs.de (M.H.); 5 Department of Food Hygiene and Environmental Health, University of Helsinki, P.O. Box 66, FI-00014 Helsinki, Finland; yagmur.derman@helsinki.fi (Y.D.); katja.selby@helsinki.fi (K.S.); hannu.korkeala@helsinki.fi (H.K.); 6 BIOTEM, Parc d’activité Bièvre Dauphine 885, Rue Alphonse Gourju, 38140 Apprieu, France; 7 LFB Biotechnologies, Therapeutic Innovation Department, 59, Rue de Trévise, BP 2006-59011 Lille Cedex, France; remi.urbain@ecdysispharma.com (R.U.); fontaynea@lfb.fr (A.F.); 8 Ecdysis Pharma, Bioincubateur Eurasanté, 70 Rue du Dr Yersin, 59120 Loos, France; 9 National Institute for Biological Standards and Control (NIBSC), a Center of the Medicines and Healthcare Products Regulatory Agency, Division of Bacteriology, Blanche Lane, South Mimms, Potters Bar, Hertfordshire EN6 3QG, UK; Thea.Sesardic@nibsc.org

**Keywords:** AntiBotABE, botulinum, toxin, phage-display, IgG, neutralization, botulism, biodefense, recombinant, oligoclonal antibodies

## Abstract

The goal of the AntiBotABE Program was the development of recombinant antibodies that neutralize botulinum neurotoxins (BoNT) A, B and E. These serotypes are lethal and responsible for most human botulinum cases. To improve therapeutic efficacy, the heavy and light chains (HC and LC) of the three BoNT serotypes were targeted to achieve a synergistic effect (oligoclonal antibodies). For antibody isolation, macaques were immunized with the recombinant and non-toxic BoNT/A, B or E, HC or LC, followed by the generation of immune phage-display libraries. Antibodies were selected from these libraries against the holotoxin and further analyzed in in vitro and ex vivo assays. For each library, the best ex vivo neutralizing antibody fragments were germline-humanized and expressed as immunoglobulin G (IgGs). The IgGs were tested in vivo, in a standardized model of protection, and challenged with toxins obtained from collections of *Clostridium* strains. Protective antibody combinations against BoNT/A and BoNT/B were evidenced and for BoNT/E, the anti-LC antibody alone was found highly protective. The combination of these five antibodies as an oligoclonal antibody cocktail can be clinically and regulatorily developed while their high “humanness” predicts a high tolerance in humans.

## 1. Introduction

The aim of the AntiBotABE Program was to develop recombinant antibodies that neutralize botulinum neurotoxins (BoNT) A, B and E, responsible for most human botulism cases. The heavy and light chains (HC and LC) of the three BoNT serotypes were targeted to achieve a synergistic effect (oligoclonal antibodies). Our strategy used macaque immune libraries to develop neutralizing/protective antibodies and was successfully applied in former projects against ricin [1], the lethal toxin of anthrax [2,3], Venezualan equine encephalits virus [4], Western equine encephelatis virus [5] and Marburg virus [6].

### 1.1. Background

BoNTs are among the most toxic known substances—whether of biological or chemical origin, they are part of the “dirty dozen” agents listed as possible bioweapons. The Gram-positive, anaerobic spore-forming bacterium *Clostridium botulinum* and other *Clostridium* spp. produce seven serotypes (A–G) of botulinum neurotoxin (BoNT), among which only four serotypes cause human botulism. In humans, botulism is characterized by a progressive and flaccid muscle paralysis that occurs naturally following food poisoning or colonization of the gastrointestinal tract by BoNT-producing *Clostridia* [7,8]. Human botulism is generally caused by BoNT/A, B and E while BoNT/F is involved in only 1% of food poisoning-related cases of botulism [9,10,11]. The BoNT/H has recently been presented as a new serotype of botulinum toxin in 2014 [12,13]. However, further analyses indicate that this is a hybrid-like BoNT, containing regions of BoNT/A1 and BoNT/F5, which is fully neutralized by serotype A antitoxin [14]. The more recently discovered BoNT/X adds to the BoNT diversity as a recognized new serotype as well as the “BoNT-like” toxin from Weissella, a homolog of BoNTs that also cleaves VAMP2 [15,16].

Botulinum neurotoxin A (BoNT/A) is the deadliest biological substance currently known, with lethal dose values (LD50: toxin dose that kills half of the animal population) of 1 ng/kg by the intravenous and subcutaneous routes and 3 ng/kg by the pulmonary route. These values were extrapolated from experiments with non-human primates. BoNT/E-related intoxications are scarcer than those related to BoNT/A and BoNT/B, but the median LD50 of BoNT/E is estimated to be as low as that of BoNT/A, equal to 1.1 ng/kg in mice and monkeys by intraperitoneal route [17]. Like a few other BoNT serotypes, BoNT/E is secreted as a unique inactive chain by group II *C. botulinum* strains that requires activation by host proteases. This process—called nicking—is associated with a 100-fold increase in toxicity [18]. Due to its extreme toxicity, ease of production and lack of antidotes, BoNT is classified as a category A biothreat agent by the United States Centres for Disease Control and Prevention [19,20]. The Soviet Union and Iraq have been suspected of weaponizing BoNTs as reported by U.N. Officers (but rejected by several countries). A former scientist in the Russian bioweapon program reported that Soviets had attempted splicing the botulinum gene into other bacteria [21,22]. Furthermore, the risk of contamination of the food chain by BoNTs has been highlighted in several potential scenarios [23]. Besides potential bioterrorist attacks, naturally-occurring food intoxications—rare but often severe—are still encountered including infant botulism cases due to intestinal colonisation by *Clostridium* spores and intoxications due to the cosmetic use of poor quality BoNT have also been reported.

### 1.2. Botulinum Neurotoxins (BoNTs)

Botulinum neurotoxins (BoNTs) cause flaccid paralysis by inducing a blockade at voluntary motor and autonomic cholinergic junctions that, if not treated, causes a deadly paralytic disease called botulism. BoNTs are type A-B heteromeric molecules composed of a 100 kDa HC and a 50 kDa LC. BoNT is a modular nanomachine composed of a N-terminal Zn^2+^-metalloprotease, which cleaves the SNAREs (Soluble *N*-éthylmaleimide-sensitive-factor Attachment Protein Receptors); a central helical protein-conducting channel, which chaperones the protease across endosomes; and a C-terminal receptor binding module, consisting of two subdomains that determine target specificity by binding to a ganglioside and a protein receptor on the cell surface, triggering endocytosis [24,25]. The HC binds to polysialo-gangliosides and, depending of the BoNT serotype, to different receptors on the surface of the motoneurons [26,27], triggering the internalization by dual-receptor-mediated endocytosis with translocation of the LC into the cytosol. The properties and mode of actions of BoNTs have paved the way for neuronal transport elucidation and protein-protein interaction while stimulating basic mechanistic studies [28]. Botulism occurs when BoNT has reached the peripheral nerve endings via the bloodstream—commonly due to food intoxication—but mechanisms behind the absorption of BoNT through mucosal membranes are not well understood. The BoNT as a complex comprises hemagglutinin proteins (HA). Epithelial cell line based assays indicate that HA proteins are pathogenic factors that disrupt the paracellular barrier of the intestinal epithelium by directly binding to E-cadherin and inhibiting E-cadherin-mediated cell-to-cell adhesion [29,30,31,32]. In addition, in a mouse ligated intestinal loop model, BoNT translocation through the intestinal barrier has been shown to occur via an endocytosis-dependent mechanism while BoNT further targeted neuronal cells and neuronal extensions in the intestinal submucosa [33,34]. However, BoNT serotypes E and F lack HA encoding genes and are produced without HA proteins, yet they cause botulism.

Despite extensive research, no small synthetic molecule has been approved for therapeutic use against botulism and Europe now relies on a heptavalent equine anti-toxin serum (HBAT, by Cangene Corporation, Winnipeg, MB, Canada, now part of Emergent Biosolutions), of which there are limited stockpiles and which still presents the risk of inducing adverse effects. Development of small inhibitors acting independently of BoNT immunological properties and targeting a common step of the intoxication process is encouraged, yet no specific inhibitor has been developed to date [35]. In the USA, two treatments based on the passive administration of antibodies are available. The first one—BabyBIG^®^—is supplied by the California Department of Public Health and is composed of human immunoglobulins obtained from pooled-plasma from subjects immunized with a pentavalent vaccine composed of BoNT/A, B, C, D and E toxoids. The second antidote consists of a preparation composed of antitoxins obtained from horses immunized with the heptavalent vaccine (HBAT) [36]. Two major limitations are that the stock of BabyBIG^®^ is limited and that the equine serum (HBAT) may cause serious adverse effects such as serum sickness [37,38]. The HBAT contains fragments of IgG targeted against seven BoNT types derived from equine plasma consisting of <2% intact IgG and ≥90% Fab or F(ab’)2 immunoglobulin fragments to reduce the hypersensitivity reaction [39]. Nonetheless, the Fab and F(ab’)2 fraction are eliminated from blood circulation more rapidly than intact IgGs, which makes HBAT plasma’s half-life shorter and may lead to serious consequences when botulism or exposure to BoNT is prolonged [40,41]. This occurred when a patient with BoNT/F intestinal botulism showed improvement after HBAT administration but bilateral descending flaccid paralysis recurred when Fab/F(ab’)2 IgG fragments were cleared from the circulation and BoNT/F rebounded [40].

BoNT is a double-edged sword with both toxic effects and therapeutic benefits, excluding vaccination as a widely applied preventative measure. Vaccines against botulism have been developed but vaccination is rarely used as its effectiveness has not been fully evaluated and its safety is questionable [41]. Thus, vaccination is only used for individuals with a high risk of exposure such as health care providers, researchers, first responders, and military personnel [42]. Meanwhile, BoNTs have been introduced as a safe and effective treatment for a wide range of disorders associated with involuntary muscle contractions and spasm disorders, and these ever-increasing medical indications prevent large scale vaccination against botulism [43]. Minute doses of this deadly poison are used therapeutically to locally paralyse muscles for clinical or cosmetic benefit. Initially used to treat strabismus, botulinum toxin has now more than a hundred possible medical applications including movement disorders, hemifacial spasm, essential tremor, tics, writer’s cramp, cervical dystonia, cerebral palsy and vascular cerebral stroke. Botulinum toxin has recently been approved for chronic pain, migraine headache, overactive bladder and inflammation [44,45].

The current situation therefore supports the need for new human-like antibody preparations that are highly-effective and better tolerated than equine antibodies but because BoNT serotypes differ by up to 70% in their amino acid sequence, it is necessary to neutralize each serotype with specific antibodies [46]. More importantly, for human application, optimal tolerance of antibodies is of major therapeutic relevance. However, it is not always possible to immunize human volunteers with the antigen of interest, particularly in the case of biological warfare agents. Human volunteers may be replaced by non-human primates for the construction of immune libraries. It was demonstrated that macaque antibodies are close to their human counterparts, which might support a good tolerance in therapy [47,48]. The AntiBotABE project focused on the development of recombinant antibodies to neutralize the most lethal types of BoNTs: A (subtypes A1), B (B2) and E (E3).

### 1.3. Antitoxin Treatment

Several mechanisms of action can be involved in the neutralization of BoNTs, depending on whether the antibodies target the HC or LC. For antibodies that target the HC, the neutralization can occur indirectly in the bloodstream by steric hindrance, impeding the binding of the toxin, or directly by inhibiting the translocation to the Hn domain of BoNTs. For antibodies targeting the LC, the neutralization can occur directly by preventing the cleavage of the SNARE complex. Several studies have previously evidenced antibodies neutralizing BoNT/A by targeting the LC or the HC but only a few of them are of human or human-like origin and they generally neutralized only one of the five BoNT subtypes [49,50,51,52,53,54,55,56]. Antibodies targeting the HC of botulinum toxins and inhibiting cell entry have also been isolated [57,58]. In a former study, recombinant antibody fragments were isolated from an immune and a non-immune human library against botulinum toxins built from a volunteer immunized with botulinum toxoid A to E, which yielded single chain Fragment variables (scFvs) with best affinities and neutralization properties compared with those obtained from the naïve library [59]. In contrast, very few antibodies neutralizing the BoNT/A LC endopeptidase activity have been described since only three antibodies of human (4LCA), macaque (2H8) and llama (Aa1) origin have been reported to date [49,50,60]. It was suggested that the recruitment of immune effectors may be involved in vivo in the BoNTs-neutralization, even if the LC is targeted [61]. It was also shown that the combination of antibodies targeting the heavy and the LCs of BoNT/A neutralize synergistically this toxin [49].

To date, immune and naïve human libraries have been shown to be useful for the isolation of antibodies neutralizing BoNT/B [49,59,62]. Kalb et al. successfully isolated picomolar antibodies cross-neutralizing BoNT/B1 and B2 by the targeting of BoNT/B LC [62]. Two of these antibodies, 2B27 and 1B10.1, seemed to inhibit the catalytic activity of some BoNT/B subtypes by interacting with the LC in vitro, determined by endopeptidase-MS. In those studies, BoNT/B1, B2, B3, B4 and B5 toxin, as a form of complexes, were neutralized in vitro by a pool of 24 fully human antibodies. These 24 antibodies were derived from in vitro affinity-improved variants, generated from antibodies initially isolated after the panning of an immune library, but their neutralization potential was not assessed in in vivo or ex vivo predictive models. A human IgG (30B) recognizing BoNT/B-HC with a high affinity (1.12 × 10^−12^ M) was previously isolated by hybridoma technology starting from the lymphocytes of human volunteers vaccinated with the pentavalent botulinum toxoid vaccine. This antibody only showed partial inhibition of BoNT/B1 in vivo as it delayed paralysis, but did not prevent mice mortality [49]. The first report of the potentially neutralizing human monoclonal antibody against BoNT/B, derived from naïve single-chain variable fragment (scFv) phage display library against BoNT/B HC domain was reported by Zhou et al. but again this report did not include in vivo or ex vivo neutralization studies, and relied exclusively on in vitro cell-based assay. A more recent study reveals that the human monoclonal antibody called 2B18 neutralizes BoNT/B by the targeting of an epitope in the Hn domain, that is conserved by several subtypes [63]. To our knowledge there is no publication that presents the neutralization of BoNT/B by human-like monoclonal antibodies targeting the LC.

Seventeen monoclonal antibodies were previously generated by immunization of BALB/c mice using a type E toxoid binding to the HC of BoNT/E [64]. Furthermore, the scFv 4E17 was isolated from human volunteers immunized with a botulinum pentavalent vaccine that binds to an epitope located at the N-terminus of the HC [63] but no recombinant human-like antibody that neutralizes BoNT/E in vivo by targeting the LC (BoNT/E-L) has been reported to date.

**Figure 1:**
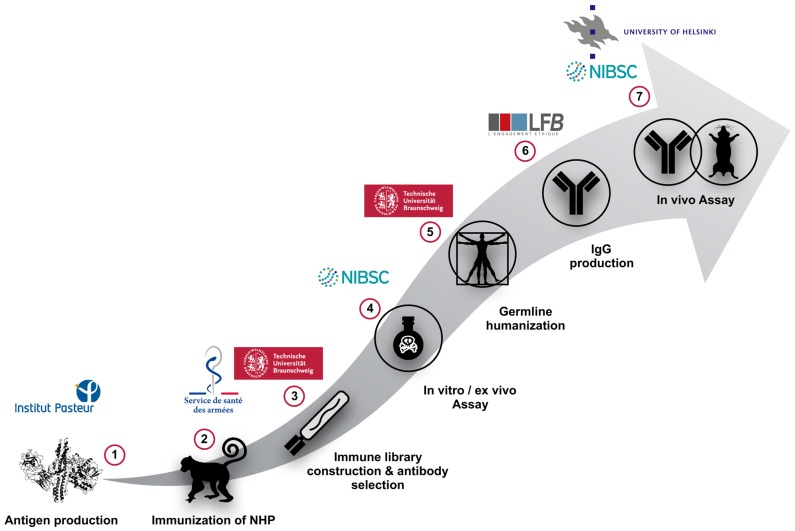
AntiBotABE Workflow.

In this review, we describe the results of the European framework project AntiBotABE (Figure 1). In this project, several recombinant antibodies neutralizing BoNT/A, B and E in an ex vivo model and in the in vivo mouse protection assay were isolated, starting from the panning of 6 immune phage-display libraries of macaque (*Macaca fascicularis*) origin.

## 2. Animal Immunization and Antibody Phage-Display Library Construction

In previous studies, macaques were already used for the generation of immune-libraries and for the isolation of antibody fragments with nanomolar and picomolar affinities. With this strategy, antibodies neutralizing fungi (e.g., *Aspergillus fumigatus)*, toxins (e.g., tetanus toxin, ricin, and anthrax) and viruses (e.g., Venezuelan equine encephalitis virus and Simian immunodeficiency virus) were isolated [1,2,4,5,6,65,66,67,68].

The standardisation of botulinum toxins used to validate animal immunization is described in detail in Section 3.2. Briefly, the effects of botulinum toxins were assessed by establishing complete dose response curves for the three main botulinum toxin serotypes on phrenic nerve-hemidiaphragm (MPNH) and titrations curves were established for standard reference antitoxins against the three major serotypes of botulinum toxins—A, B and E [69,70].

### 2.1. Immunization of Macaques (Macaca fascicularis) Using the Non-Toxic and Recombinant HC or LC of BoNT/A, B or E

Several studies compared the nucleotidic and peptidic sequences of the variable domains of human and non-human primate antibodies. These studies revealed a close proximity between human, macaque and chimpanzee antibodies. As it was observed that there is no significant difference between macaque and chimpanzee antibodies, macaques represent a model of choice for the development of therapeutic antibodies [47,48,71,72]. In the context of the AntiBotABE project, the strategy was based on the immunization of macaques because it is easier to obtain with immune-libraries, high-affinity antibodies specific to epitopes spanned on the whole immunogen [51].

All animal studies presented in this section were given specific approval from the Institut de Recherche Biomédicale des Armées Ethics Committee (Comité d’éthique de l’Institut de Recherche Biomédicale du Service de Santé des Armées) under authorization No. 2008/03.0 and were performed in accordance with all relevant French laws and ethical guidelines.

Six macaques were immunized four times each with the recombinant and non-toxic HC or LC of BoNT/A1, B2 or E3 [48]. This protocol led to a stable and high titer against the corresponding immunogen, as determined by ELISA. For the anti-BoNT/A1-LC antibodies, a titer of 1/100,000 was observed against the BoNT/A1 holotoxin [50]. For anti-BoNT/A1-HC antibodies, a titer of 1/800,000, 1/1,310,000 and 1/40,000 was observed in ELISA against BoNT/A1-HC (the immunogen), BoNT/A1 (holotoxin) and BoNT/A2 (complex), respectively. For anti-BoNT/B-LC antibodies, a titer of 1/100,000 (BoNT/B2-HC immunization) or 1/300,000 (BoNT/B2-LC immunization) was observed against the immunogen. For anti-BoNT/E-HC antibodies, a titer of 1/600,000 against BoNT/E3 holotoxin was observed. Finally, for anti-BoNT/E-LC antibodies, a titer of 1/600,000 against BoNT/E3 holotoxin was observed [51,73,74,75].

After the last antigen administration, the macaque bone marrow was iteratively sampled over a period of up to 20 days, in order to observe an optimal amplification of the DNA encoding the Fd fragments and the κ LC. At the time of optimal DNA amplification, the retro-amplified DNA was retained and used for the generation of the antibody immune libraries.

### 2.2. Phage-Displayed Immune Libraries Construction

Immune-libraries were generated as previously described [1,2,4,65,71,72,73]. Briefly, the DNA encoding the Fd fragments and the κLC were separately sub-cloned in pGEM^®^-T vector and then sequentially cloned in the pHAL14 or pHAL32 phagemid vector. These phagemid vectors allow the expression of scFv-pIII fusion protein at the surface of the phage particle. As they contain both bacterial and phagemid origins of replication, they can replicate as single and double strand phagemids and they can directly be used for phage-display screening. Each library was packaged in M13KO7 or hyper-phage. The immune-library directed against BoNT/A1-LC consisted of 3.2 × 10^8^ independent clones with a full-size insert rate of 80%. This library was packaged with M13K07 or Hyperphage [51]. The immune-library directed against BoNT/A1-HC consisted of 5.45 × 10^7^ unique clones and was finally packaged in M13K07 helper-phage [73].

The immune-library directed against BoNT/B2-HC consisted of 1 × 10^8^ independent clones and was finally packaged in M13K07 helper-phage. The immune-library directed against BoNT/B2-LC consisted of 1.1 × 10^8^ unique clones and was finally packaged in M13K07 phage [74]. The immune-library directed against BoNT/E3-LC consisted of 9.6 × 10^8^ independent clones with a full-size insert rate of 88%. This immune library was packaged with M13K07, resulting in phage titers of 3.4 × 10^13^ cfu mL [75].

### 2.3. Selection of scFv Directed against BoNT/A1 and A2, or B1 and B2 or E3 Subtypes/Serotypes by Multi-Step Panning and Screening by ELISA

A 3 rounds-panning against holotoxin BoNT/A1 or recombinant BoNT/A-LC was performed with Hyperphage and M13K07 packaged antibody-phage libraries. For the Hyperphage packaged library, 6.2 × 10^3^ antibody-phages were isolated against the BoNT/A1 holotoxin after the third and last round of panning. Using the library packaged in M13K07 phage, 1.3 × 10^5^ antibody were isolated against the BoNT/A1. For the panning against the LC, only the M13K07 packaged library was used and resulted in 5.5 × 10^8^ antibody-phages after the third round of panning. After the last round of screening, reactivity against BoNT/A1 was analyzed by ELISA on randomly hand-picked antibody clones. About 100 clones from each panning were expressed as soluble scFv for screening by ELISA. The binding of those scFv to BoNT/A1 holotoxin and BoNT/A1-LC were tested by antigen ELISA (data not shown). In total, 69 ELISA positive scFv clones binding to BoNT/A1 were identified. These clones were sequenced and 22 unique scFvs were revealed suggesting that these scFv may target different epitopes.

For BoNT/A1 HC, the phage-displayed scFvs library was subjected to 3 rounds of panning against BoNT/A1 holotoxin with a doubling number of washes after each round (5, 10 then 20 washes). After each round, only the phage having interacted with BoNT/A1 were eluted. Between the first and the second round of panning the number of eluted phage decreased from 115,000 (round 1) to 10,000 (round 2), underlining the selection of the small proportion of reactive phage among the whole immune-library. The reactivity of the phage eluted after each round against BoNT/A1 was assessed in phage-ELISA. The signal increasing 9-fold between round 1 and 3 confirmed the enrichment in scFvs highly reactive against the target [73].

To isolate scFvs cross-reacting with BoNT/B1 and B2, the hyper-immune libraries were subjected to an innovative two-time panning process (classically performed against one immunogen). The panning was initiated with the holotoxin BoNT/B1 to select scFvs reacting against the fully active toxin, which is also derived from a different subtype than the subtype used as the immunogen. First, a three-round panning was performed on BoNT/B1 and then a fourth and last round was performed against BoNT/B2 to isolate only the cross-reactive antibodies. During the panning, the number of washes was doubled between the first three rounds and unchanged during the fourth round. One hundred clones were randomly handpicked after the fourth round of panning against BoNT/B2-HC, and then sequenced to express the non-redundant and non-recombined scFvs cross-reacting with BoNT/B1 and BoNT/B2. Ten different scFvs were identified, expressed as soluble scFvs, purified and their affinities for BoNT/B1 and BoNT/B2-LC were measured. The multistep panning approach was validated because the number of eluted phage was comparable to that reported in previous studies and sub-nanomolar scFvs were produced [1,74].

For the library directed against BoNT/E LC, a multi-step panning of four rounds was performed against BoNT/E3 holotoxin (5, 10, 20 and 40 washes). After each round of panning, the reactivity of the eluted phage against BoNT/E3 was assessed by phage-ELISA and the signal increased 1.5-fold between rounds 1 and 4. After the fourth round of panning, 100 scFv-clones were randomly chosen and the scFv encoding DNA was extracted and sequenced. Eighty-five unique scFvs were observed and their affinity for in vitro-activated BoNT/E3 was measured [75].

### 2.4. Further Characterization by Affinity Measurements

Affinity was determined by surface plasmon resonance (SPR, Biacore^®^, GE Healthcare Europe Velizy-Villacoublay, France). For the LC of BoNT/A, the affinity of the 22 isolated-scFv was determined. The majority of them were in the nanomolar range, underlining a high specificity. Three out of 4 scFvs had a sub-nanomolar range affinity [51]. For anti-BoNT/A1-HC antibodies, the affinity of sixty-four scFvs was measured against BoNT/A1 and they ranged from 1.3 nM (A1HC49) to 50 nM. The affinity of the scFv A1HC38 was measured at 1.9 nM with Kon = 6.34 × 10^4^ M^−1^ S^−1^ and Koff = 1.2 × 10^−4^ S^−1^. The twenty-four scFvs (37.5% of all non-redundant and non-recombined scFvs) presenting affinities better than 10 nM were selected for the neutralization capacity assessments [73].

For the HC of BoNT/B, 10 different scFvs were expressed in *E. coli* strain HB2151 as soluble scFvs and their affinities were measured by SPR. The affinities ranged from 2.29 nM (B2-6) to 14.1 nM (B2-63) for the holotoxin BoNT/B1 and from 0.2 nM (B2-119) to 11 nM (B2-29) for BoNT/B2-HC with affinities against BoNT/B2-HC (the immunogen) always being more favourable than against BoNT/B1. The affinity of scFv B2-7 was 5 nM and 0.6 nM, for the holotoxin BoNT/B1 and BoNT/B-HC, respectively. For the LC of BoNT/B, affinities for BoNT/B1 ranged from 0.4 nM (BLC3) to 17 nM (BLC18) for BoNT/B1. Affinities for BoNT/B2-LC were generally less favourable than for the holotoxin BoNT/B1 [74].

The affinity was measured only for the four anti-BoNT/E3 antibodies that were found inhibiting in the endopeptidase assay. Their affinity ranged from 0.01 to 9.16 nM [72]. In addition to the affinity measurements of the selected scFvs, affinity of the germline-humanised IgGs was also measured by SPR but the data are currently classified by the European Commission [76].

## 3. In Vitro Evaluations and Ex Vivo Neutralizing Properties of High Affinity scFvs and scFv-Fcs

**Figure 2:**
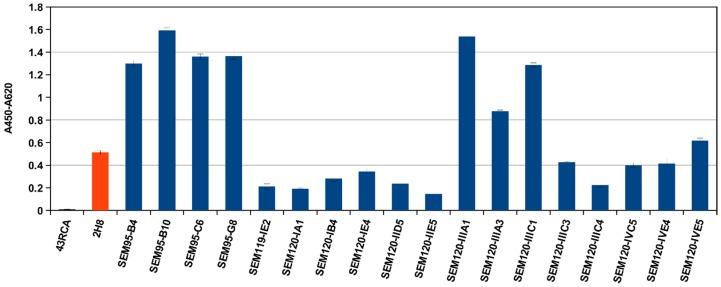
ELISA on immobilized BoNT/A LC. 1 μg of the 19 selected binders against BoNT/A1 tested as scFv-Fc fusion (murine Fc) on 100 ng directly immobilized BoNT/A LC. Modified figure from our previous publication: Miethe et al. 2014 [51].

Antibodies targeting the HC are suspected to block the binding of the toxin to the cell surface, inhibiting the penetration of the toxin into the cell or the translocation of the LC through endocytosis vesicles into the cytosol. The antibodies targeting the LC may also have the same action, but only indirectly by steric hindrance. In addition, antibodies targeting the LC are considered to directly inhibit the catalytic activity of the light chain. To fully explore the neutralizing capacities, two different approaches were chosen for the AntiBotABE project: an in vitro endopeptidase approach to explore the inhibition of the catalytic activity by antibodies targeting BoNTs LC and the ex vivo approach to evidence a broader neutralizing action of the antibodies by preventing the binding and translocation of the BoNTs. The scFvs initially found to be binding BoNTs were converted into scFv-Fc antibodies for the assessment of their neutralizing properties. For example, a murine IgG2C-Fc was used for the anti-BoNT/A1 binders, resulting in a chimeric antibody. Nineteen of the 23 anti-BoNT/A1 binders were re-cloned and produced as scFv-Fc with a murine IgG2c-Fc part. The yield of the remaining four antibodies was too low for further analysis. The antigen binding of the 19 scFv-Fc was compared and validated by ELISA on immobilized BoNT/A LC before in vitro and ex vivo evaluations (Figure 2).

### 3.1. In Vitro Screening of Anti-BoNT LC Antibodies as scFv or scFv-Fc by Endopeptidase Inhibition Assays

The in vitro endopeptidase immunoassay described in a previous study for the BoNT/A toxin was optimised to assess the inhibitory properties of scFv-Fc preparations directed against BoNT/A, B and E LCs [50]. The curve obtained in this assay yields an ELISA signal as a function of BoNT/A, B or E endopeptidase activity in which a 50% decrease in signal strength (within the linear part) coincides with a 50% decrease in endopeptidase activity.

Nineteen antibodies directed against the LC of BoNT/A were produced as scFv-Fc in mammalian cells and were tested against BoNT/A1 in vitro. The results of endopeptidase assays revealed that 8 antibodies gave higher levels of endopeptidase inhibition than the 2H8 antibody isolated in the initial study [50]. With regard to the scFv-Fc concentration inhibiting 50% of BoNT/A1 in vitro, the SEM120-IIIC4 gave the highest level of inhibition. The molar ratio of antibody to toxin at which 50% inhibition of endopeptidase activity was found to be almost 0.5:1 for the SEM120-IIIC4 antibody. All antibodies inhibiting endopeptidase more efficiently than 2H8 and with little or no background signal were selected for further ex vivo studies [51].

The molar ratio (antibody to toxin) at which a 50% inhibition of endopeptidase activity was observed was assessed for all tested antibodies to compare their inhibitory profiles. The molar ratio for the 2H8 produced as a scFv was 64,000:1, [51] while, a 50% inhibition was observed at a molar ratio of 6500:1 for the 2H8 when produced in the scFv-Fc format, presumably due to an avidity effect. Nevertheless, the best molar ratios were those obtained for SEM120-IIIC4 (0.5:1), SEM120-IID5 (1:1) and SEM120-IIIC1 (5:1) as scFv-Fc antibodies. Those molar ratios were far more favourable than for the human 4LCA IgG reported previously, yielding 63% inhibition of BoNT/A endopeptidase activity with a corresponding molar ratio of 10,000:1 [49] and for the llama antibody Aa1 exhibiting a 50% inhibition only against the LC of BoNT/A and with a molar ratio of 200:1 [60].

The in vitro endopeptidase assay was performed against both BoNT/B1 and BoNT/B2 subtypes in order to select cross-neutralizing scFvs directed against the BoNT/B2-LC. Due to their low nanomolar affinities, 26 scFvs were assessed for their inhibition properties in the VAMP2 endopeptidase assay. Six (23%) scFvs inhibited the catalytic activity of 0.2 LD50 mL^−1^ complex BoNT/B2, and only two of these (7.6%) also inhibited 30 LD50 mL^−1^ of pure BoNT/B1 subtype. The inhibition of BoNT/B1 and BoNT/B2 catalytic activity observed for all scFvs was dose-dependent and considerably improved when the more stable scFv-Fc bivalent format was used since it is more similar to the IgG format. This observation was consistent with our previously published studies showing a reduced inhibition ratio with scFv-Fcs antibodies targeting BoNT/A-LC in comparison to the corresponding scFv format. In addition, similar findings were obtained for the antibody targeting the BoNT/E-LC [75].

The 38 selected scFv targeting BoNT/E3 were also assessed for their inhibition capacities in the SNAP-25 endopeptidase immunoassay. The best scFv: ELC76 inhibited the endopeptidase activity of BoNT/E3 at a molar ratio of scFv to toxin of 14,765:1 (IC50 of 33.8 nM). The scFvs inhibiting the toxin endopeptidase activity were selected for further analyses using the ex vivo MPNH assay and affinity measurements.

### 3.2. Ex Vivo Screening of Anti-BoNT HC and Anti-BoNT LC Antibodies as scFv or scFv-Fc in the Mouse Phrenic Nerve Hemidiaphragm Paralysis Assay

Preparation of standardized botulinum toxins and anti-toxins: Purified type A1 and B1 hemagglutinin free toxins were purchased from Metabiologics Inc. (Madison, WI, USA) and diluted to 20,000 LD50 mL^−1^ in gelatin (0.2% *w*/*v*) phosphate buffer (GPB). Concentrated pure hemagglutinin free botulinum type E3 from Metabiologics was trypsinised and diluted in GPB, pH 6.5. Purified type A2 hemagglutinin free and complex toxins were purchased from Metabiologics Inc. (Madison, WI, USA) and diluted to 500,000 LD50 mL^−1^ in GPB pH 6.5. A commercially available equine polyclonal F(ab’)2 preparation (Novartis Vaccines, Marburg, Germany) was used as a positive control in neutralization assays.

For standardization of the BoNTs, the effects of botulinum type A, B and E toxins on MPNH were assessed by establishing complete dose response curves for the three main botulinum toxin serotypes. The use of Balb/c in-bred mice greatly improved the standardization of the hemidiaphragm assay due to very low variability in the 50% paralysis time measurements whatever the dose or serotype of the botulinum type. In addition, the increased precision of the assay using in-bred mice allowed more accurate evaluations of toxin or neutralizing antitoxin levels.

Additional work was performed with botulinum toxin subtypes A2 and B2 to establish the dose paralysis curve in order to test new scFvs directed against A2 and B2 subtypes. Titration curves were established for standard reference antitoxins against the three major serotypes of botulinum toxins A, B and E. Series of monovalent standard antitoxins and polyvalent licensed and experimental products were previously tested which confirmed excellent correlation between the ex vivo MPNH assay and the in vivo mouse bioassay.

The preliminary in vitro evaluations of selected antibodies had to be completed with ex vivo neutralization capacity assessments in order to assess their neutralizing potential in subsequent in vivo studies. In the MPNH assay, the in vivo respiratory paralysis caused by BoNT is closely reproduced ex vivo in mouse hemi-diaphragmatic tissues [69]. In the neutralizing activity assessment of new antibodies against the main toxin serotypes A, B and E, this ex vivo assay approach provided results highly consistent with those of the mouse in vivo assay [70].

In the MPNH ex vivo model, the time to reach 50% paralysis after the addition of the mixture of botulinum toxin/scFv-Fc is assessed by fitting to the linear part of the paralysis curve. The neutralization potential of the scFv-Fc antibodies is directly correlated with their ability to prolong the 50% paralysis time by delaying BoNT-induced paralysis onset. A greater antibody neutralizing potency is associated with a longer time period required to reach 50% paralysis of the hemidiaphragm tissue while exposed to the same dose of toxin.

**Figure 3:**
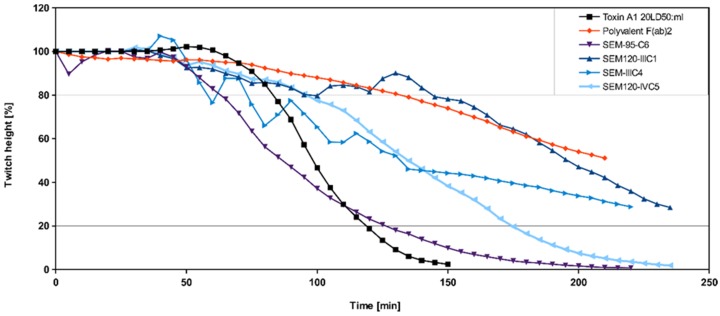
Neutralization of BoNT/A1 by scFv-Fc directed against BoNT/A LC on the mouse phrenic nerve-hemidiaphragm. Modified figure from our previous publication: Miethe et al. 2014 [51].

To confirm the validity of the neutralization properties of the antibodies showing inhibitory properties against BoNT/A LC in the endopeptidase assay, the ex vivo neutralization capacity of eight inhibitory antibodies was tested using 30 µg/mL of each in the scFv-Fc format. The positive control was a trivalent equine F(ab’)2 polyclonal serum and the negative control was a anti-ricin antibody 43RCA. Five of the eight antibodies initially selected (SEM120-IID5, SEM120-IIIC1, SEM120-IIIC4, SEM95-G8 and SEM120-IE4) strongly neutralized BoNT/A1 by significantly delaying the BoNT/A1 toxin induced paralysis. Each of the five antibodies doubled the 50% paralysis time (50% PT), i.e., 202 min, 200 min, 188 min, 181 min and 173 min for SEM120-IID5, SEM120-IIIC1, SEM120-IIIC4, SEM95-G8 and SEM120-IE4, respectively. Moderate levels of neutralization of the same toxin were obtained in the hemidiaphragm assay for the SEM119-IE2 (50% PT: 164 min) while the SEM120-IVC5 displayed weak neutralization ability (50% PT: 116 min) (Figure 3). For future immunotherapeutic purposes, the best antibody was produced as a complete germline humanized IgG.

For the anti-BoNT/A HC antibodies, all scFvs with affinities better than 10 nM against BoNT/A1 HC were tested in the ex vivo MPNH assay, to identify those neutralizing BoNT/A1 holotoxin and cross-neutralizing BoNT/A2 toxin complex. The best scFv cross-neutralizing BoNT/A1 and BoNT/A2 was then finely analyzed against BoNT/A1 and BoNT/A2, in decreasing doses of the scFv. The 50% paralysis time with BoNT/A1 was delayed by 115 min in presence of 20 mIU mL^−1^ of positive control. Among the 24 scFvs tested, 3 efficiently neutralized BoNT/A1 at 20 LD50 mL^−1^ at the concentration of 27.25 μg mL^−1^ (A1HC17), 5.21 μg mL^−1^ (A1HC38) or 12.25 μg mL^−1^ (A1HC45). These scFvs delayed the 50% paralysis time by more than 145 min (145%), by 164 min (164%) and by 95 min (95%), respectively. The three scFvs neutralizing BoNT/A1 were then tested at a high concentration against BoNT/A2 in the complex form. At concentrations of 20 μg mL^−1^ (A1HC17) or 15 μg mL^−1^ (A1HC38 and A1HC45), the 50% paralysis time was delayed by 115 min (97%, A1HC17), more than 220 min (188%, obtained by extrapolation, A1HC38) and 45 min (38%, A1HC45) [73].

As the scFv A1HC38 was the most efficient cross-neutralizing scFv, it was selected for further characterization at lower dilutions against BoNT/A1 and BoNT/A2. At concentrations of 0.5 and 1 μg mL^−1^, A1HC38 delayed the 50% paralysis time by 45 and 120 min, respectively (45% and 120% delay in paralysis, respectively). The minimum neutralizing concentration was considered to be close to 0.5 μg mL^−1^. At concentrations of 7 and 5 μg mL^−1^, A1HC38 delayed the paralysis induced by BoNT/A2 by more than 227 min (more than 192% delay in paralysis) and by 65 min (55% delay in paralysis), respectively [73].

These results are quite promising since efficient neutralization of BoNT/A usually requires the association of several neutralizing antibodies and because neutralization properties of the A1HC38 were even stronger than with the polyclonal F(ab’)2 antitoxin. Antibodies targeting the HC could neutralize the toxin by different mechanisms, mainly by preventing toxin binding and its internalization into the motor neurons, but they also could indirectly inhibit the catalytic activity, by steric hindrance. The affinity of A1HC17 and A1HC38 were measured and were equal to 4.79 nM and 1.9 nM, respectively, and it is interesting to note that no strict correlation between high affinity and neutralization properties was observed since the four best scFvs in terms of affinities were not neutralizing.

The scFvs BLC3 and BLC42 were also tested in the ex vivo MPNH assay, but their neutralization capacities were found to be weak (data not shown). However, the use of a more stable version of the BLC3 (scFv-Fc) resulted in much stronger neutralization of pure BoNT/B1 (100 LD50 mL^−1^) as well as complex BoNT/B2 (0.2 LD50 mL^−1^). Neutralization of BoNT/B2 subtype, but not of BoNT/B1, by scFv BLC3 was better than that for the commercial polyvalent F(ab)2 antitoxin, at 20 mIU mL^−1^, which increased 50% paralysis time by about 100 min for both subtypes.

Initial screening of all the anti-BHC scFvs in the MPNH assay identified 6 scFvs with some inhibitory properties against BoNT/B1 (100 LD50 mL^−1^), with scFv B2-7 showing the strongest neutralization at 29 μg mL^−1^ (comparable to that observed with commercial polyvalent F(ab)2 antitoxin at 20 mIU mL^−1^). B2-7 was thus selected for further characterisation against 0.2 LD50 mL^−1^ of complex BoNT/B2 (equipotent paralytic dose to 100 LD50 mL^−1^ of pure BoNT/B1). The dose response for neutralization of complex BoNT/B2 by scFv B2-7 showed a 52 min delay of the 50% paralysis time, representing 23% inhibition at 5.0 μg, which increased to a 374 min delay and 73% inhibition at 10 μg of scFv B2-7.

**Figure 4:**
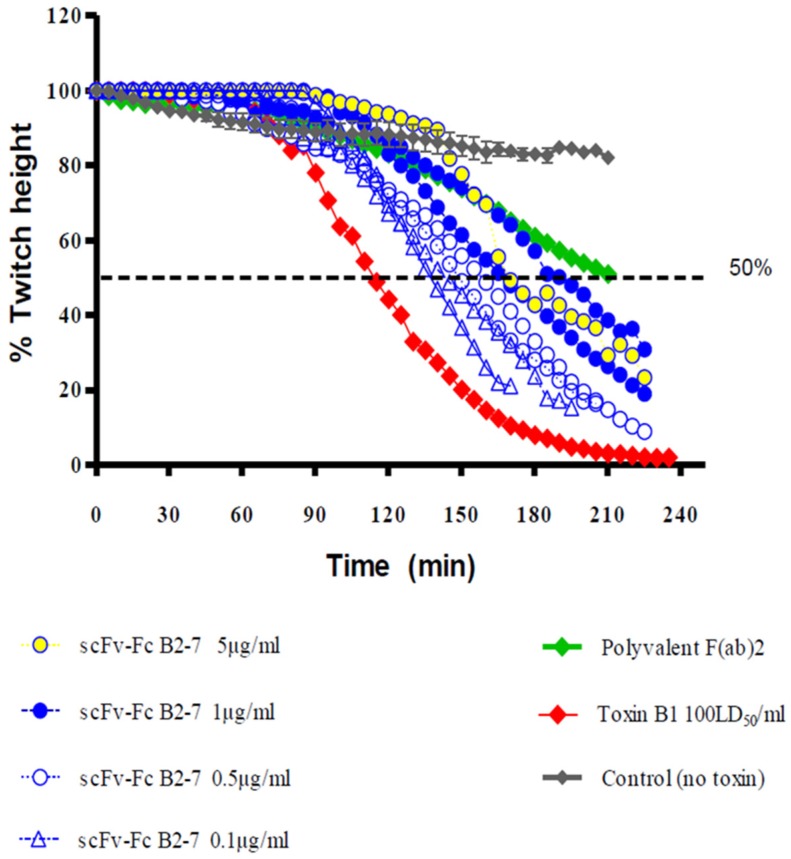
Neutralization activity in MPNH of scFv-Fc B2-7 targeting BoNT/B-HC. Modified figure from our previous publication: Rasetti-Escargueil et al. 2015 [74].

Strong neutralization was also confirmed with the scFv-Fc fusion form of the B2-7 against the same concentrations of BoNT/B1 and BoNT/B2 subtypes, inducing a 67 min and 80 min prolongation of the 50% paralysis time at 1.0 and 30 μg mL^−1^, respectively. Hence, the B2-7 is the most promising candidate for further development targeting the HC of BoNT/B [74] (Figure 4).

For the anti-BoNT/E antibodies, to confirm that the antibodies inhibiting the endopeptidase activity of BoNT/E3 in vitro do have toxin neutralization properties, their neutralization capacity against BoNT/E3 was tested in the ex vivo MPNH assay. Only ELC18, ELC51 and ELC76 were tested ex vivo [75].

When the same three scFv were tested for neutralization of 20 LD50 mL^−1^ of pure BoNT/E3 (330 pg/mL) in the MPNH, the scFv ELC18 was identified as most neutralizing (445 min paralysis time) and further lower doses were studied. Significant neutralization (at least 25% inhibition of activity, 164 min paralysis time) was confirmed with as low as 0.3 nM of scFv ELC18, and almost 50% inhibition observed at ~3.3 nM. This represents a more favourable neutralization profile compared to in vitro inhibition with molar ratio of ~1500:1 (an increase of 33-fold). For further analysis ELC18 was converted into the scFv-Fc antibody format and tested at different dilutions in the ex vivo paralysis assay where at lowest concentration of 0.9 nM a 50% paralysis time of 237 min was achieved and represents a molar ratio (scFv-Fc:toxin) of ~393:1. See the summary table demonstrating affinity for the in vitro endopeptidase (for LC only) and ex vivo studies results for selected scFv or scFv-Fc antibodies (Table 1).

Regarding BoNT/E3 HC, ex vivo screening of the 40 scFvs in the hemidiaphragm paralysis assay did not identify any scFvs with inhibitory properties against BoNT/E3 toxin when 20 LD50 mL^−1^ (330 pg/mL) of pure E3 toxin was used. Potential synergistic effects were also assessed: twenty-five of non-competing scFvs were identified, paired and tested, but they did not show neutralizing capacities against BoNT/E3 while the positive control polyclonal antibody F(ab)2 strongly inhibited BoNT/E3 at 20 mIU/mL in the ex vivo MPNH (data not shown).

### 3.3. Isolation of the Most Promising Antibody Combinations (Anti-BoNT/HC and Anti-BoNT/LC Directed against the Same Serotype) Using the In Vivo Flaccid Paralysis Assay, Synergistic Protection Assessment, Epitope Characterization and Affinities Measurements

**Figure 5:**
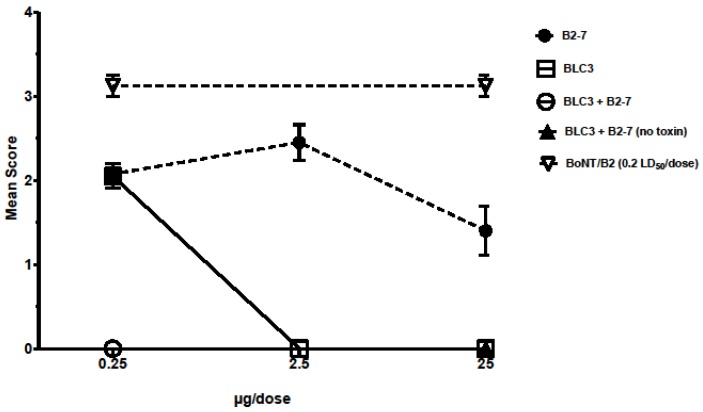
Neutralization activity of scFv-Fc BLC3 and B2-7 in vivo. Modified figure from our previous publication: Rasetti-Escargueil et al. 2015 [74].

The BLC3 and B2-7, targeting BoNT/B-LC and HC respectively, were tested in scFv-Fc forms in the mouse in vivo flaccid paralysis model—both individually and in combination for a synergistic neutralizing effect—against complex BoNT/B2 (0.2 LD50 per dose). Complete protection was achieved with 25 and 2.5 μg of BLC3, whereas B2-7 did not fully protect mice at 25 μg per dose, but lowered the intensity of paralysis. Those neutralization results were comparable at both 24 h and 48 h observation times. Additional neutralization studies with higher concentrations (50 μg and 100 μg) of scFv B2-7 demonstrated a similar effect with no benefit of dose increase. However, the combination of BLC3 and B2-7 fully protected mice in vivo where protection was not observed by each antibody alone (0.25 μg of each antibody) confirming effective and substantial synergistic effect [74] (Figure 5).

**Figure 6:**
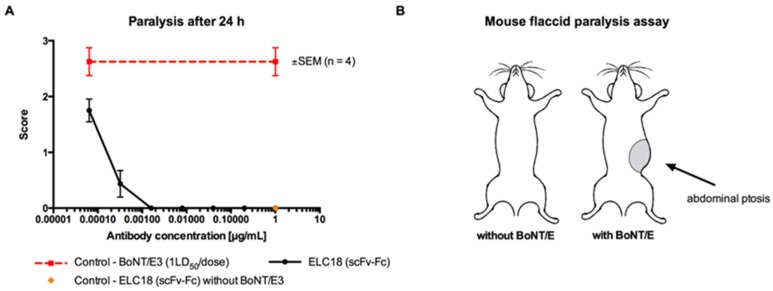
In vivo mouse paralysis assay with ELC18 as scFv-Fc. Modified figure from our previous publication: Miethe et al. 2015 [75].

The scFv-Fc form of ELC18 targeting BoNT/E-LC was also tested in vivo in the non-lethal mouse flaccid paralysis protection model against pure BoNT/E3 (1 LD50 per dose). Complete protection was achieved with 1.6 ng of ELC18, this represents a molar ration (antibody:toxin) of ~87:1 [75] (Figure 6).

## 4. Germline-Humanization and In Vivo Characterization of the Selected Antibodies

The development of neutralizing human-like scFv-Fcs targeted against LCs and HCs of BoNT/A, B and E has been described in our previous studies [51,73,74]. However, for future medical applications optimal tolerance in humans has to be ensured when using such antibodies and several humanization methods have been described [77]. One method especially for antibodies derived from non-human primates (NHP)—germline-humanization—is based on the modification of the NHP antibody framework regions (FR) to increase the level of identity with the FRs encoded by the closest human germline gene sequences [78]. Germline humanization consists of series of mutations in the framework regions (FRs) of non-human primate (NHP) antibody to obtain humanized FRs while preserving high epitope affinity and neutralization capacity. It has been shown that human germline FRs of IgM antibodies are better tolerated by the immune system than FR sequences derived from IgG antibodies, which carry somatic hypermutations resulting from affinity maturation that probably form immunogenic sequences [79,80].

### 4.1. Generation of Variants of the Germline Humanized Antibodies and Identification of the Most Promising Germline-Humanized Variant for Each Library

For optimal immune tolerance in humans, the framework regions of macaque antibodies should be very similar to the corresponding human germline sequences. The potential immunogenicity of antibodies can be estimated by calculating the level of identity of the corresponding framework regions to the most similar human germline-encoding framework regions. This identity level of antibodies is also known as the germinality index (GI) [3].

In our previous studies, we reported the generation of neutralizing scFv and scFv-Fc derived from macaques against BoNT/A, BoNT/B and BoNT/E: SEM120-IIIC1 (anti-BoNT/A LC), A1HC38 (anti-BoNT/A HC), BLC3 (anti-BoNT/B LC), B2-7 (anti-BoNT/B HC) and ELC18 (anti-BoNT/E LC) [51,73,74]. Based on the physicochemical classes of the amino acids, differences in the FRs were classified as very similar, similar, dissimilar or very dissimilar AA according to IMGT [81].

Twenty-two scFv isolated by panning for isolation of anti-BoNT/A1 LC antibodies were sequenced by GATC Biotech (Konstanz, Germany) and compared with human germline sequences, with the IMGT/V-QUEST online tool from the International ImmunoGeneTics information system^®^ (IMGT, Montpellier, France) [82]. This tool identifies the human germline sequences most similar to any given variable region and calculates the germinality index (GI), defined as the percentage identity between a given framework region (FR) and the most similar human germline sequence. GI indirectly predicts the tolerance of a VH or VL sequence, based on the assumption that germline encoding sequences are the best tolerated, as they are part of the IgM immunoglobulins. The GI values of the 23 scFv ranged from 79.1% to 92.3% for VH and from 77.5% to 92.1% for VL. For comparison, the mean GI values of 100 unpublished human scFv from a naïve scFv library (HAL7/8) 43 were 96.6% for VH and 94.8% for VL. The germline humanization of macaque antibodies is a potentially promising method for increasing immune tolerance for human treatment. An analysis of the human germline genes most similar to the genes encoding the 22 selected scFv in addition to 2H8 showed that VH and VL sequences were highly diverse. The germinality index (GI) for each VH and VL was calculated and provided an indication of the identity between framework regions of the selected scFv and those encoded by the most similar human germline genes, as a percentage [51].

After the isolation and the sequencing of the scFvs targeted against HC of BoNT/A1, a computational analysis using IMGT/V-QUEST tool was performed to retrieve the human germline sequences closer to the sequence of the 24 selected scFvs. The use of three IGHV family genes (families IGHV-1, -3 and -5) was observed. These VH were combined to four different IGHJ family genes (families IGHJ-2, -4, -5 and -6). Regarding LCs, the use of two different IGKV genes (1 and 3) was observed: IGKV1-39*01 and IGKV1-16*01 (5 occurrences each), IGKV1-17*01 (4 occurrences), IGKV1-27*01, IGKV1-13*02 and IGKV1-9*01 (2 occurrences each) and finally one occurrence of IGKV1D-13*01, plus three single occurrences of IGKV3 (IGKV3-7*02, IGKV3-11*01 and IGKV3-20*01). These IGKV genes are also combined to different IGKJ family genes (families IGKJ-1, -2, -3 or -4). The GI of the 24 best scFvs ranged between 81.13% and 87.72%, underlining their high identity level with human sequences and thus their likely low immunogenicity. The G-score is another parameter that could indirectly predict the tolerance of the scFv, but it is based on the comparison with the expressed genes and not with germline genes [71,72]. The G-scores of the 24 selected scFv were also determined and ranged between −1.01 and −2.37. Even if the G-score of the LCs of A1HC34, A1HC45 and A1HC65 were positives then all mean G-score were negatives.

For comparison, the mean GI values of 100 unpublished human scFv from naive antibodies libraries are 96.6% for VH and 94.8% for VL [51]. The mean G-score of A1HC17 and A1HC38 were −1.53 and −1.75, respectively, thus they were respectively “as human” as 6% and 4% of the human antibodies belonging to the same germline gene family present in the Kabat database. A sequence with a G-score equal to zero has the same identity level with human expressed antibodies than the average identity observed when human antibodies are compared between them. A sequence with a negative G-score presents lower than average identity level and should be humanized to increase its tolerance. Because A1HC17 and A1HC38 cross-neutralized the BoNT/A1 and BoNT/A2 sub-types, they are good candidates for a future clinical development, especially in combination with the anti-LC antibody SEM120-IIIC1.

For anti-BoNT B HC antibodies, the IMGT/V-QUEST tool was used to analyse the DNA sequence of the isolated scFv to identify the V, D and J genes from which they would have derived if they had been of human origin. The percentage of similarity between the framework regions of each scFv peptide sequence and the most similar human germline peptide sequences (percentage referred to as Germinality Index, GI) was calculated for each isolated scFv and averaged (average of the antibody heavy and LC of the framework regions of each scFv). For the 10 isolated scFvs, GIs ranged from 80 % to 89% and the mean GI was 82.7%, which highlights their human-like character. The G-score, which evaluates the “humanness,”and thus predicts scFv potential immunogenicity, was calculated for each VH and VL region and averaged for each scFv. The scFv mean G-scores for the 10 selected scFvs ranged from −0.60 to −1.87, with a mean value equal to −1.45. Similarly, for the library directed against BoNT/B2-LC, sequences identified after clones sequencing were subjected to a computational analysis. The GIs of the 26 isolated scFvs ranged from 75.82% to 87.91% for the antibody HCs and from 79.77% to 84.09% for antibody LCs, resulting in an average value for the whole scFv molecule of between 77.79% and 86%. The mean G-scores for 26 selected scFvs ranged between −1.0035 and −2.746, underlining that some scFvs are close to human antibody sequences.

After sequencing of scFvs targeting BoNT/E3 LC, analysis using IMGT/V-QUEST tool was performed, to retrieve the human germline V, (D), J genes most similar to the 38 selected scFv that were chosen for further in vitro analysis. Almost all HC V genes were dominated by IGHV4 gene family, with exception of 2 occurrences of IGHV1 and IGHV3 gene family. These genes were combined with representatives of all IGHD genes and IGHJ genes. The diversity of the LC was limited due to the presences of mostly IGKV1 genes with only one occurrence of IGKV7. These genes were combined with nearly all IGKJ genes. The Germinality Index (GI) for each VH and VL were calculated using IMGT/DomainGapAlign and provided an indication of the identity between framework regions of the selected scFv and those encoded by the most similar human germline genes, as a percentage. The average GI-values for the all 38 scFv were 86.9% (VH) and 89.4% (VL). Out of the three neutralizing scFv, ELC76 was the most human-like antibody fragment with a mean GI equal to 91.2% (87.9% for VH and 94.4% for VL) followed by ELC18 with 87.8% (85.7% for VH and 89.9% for VL). For comparison, the average GI value of 500 scFv isolated from the human naïve antibody gene library HAL7/8 [83] was 95.7%. The high humanness of the isolated antibodies against BoNT/E3 predicts a high tolerance as is the case with previous studies using the chimeric antibody lumiliximab, consisting of the variable regions of a macaque combined to human constant regions and showing high tolerance [84,85]. Table 2 demonstrates the GI% change for the five selected antibodies.

### 4.2. Expression of the Selected Germline-Humanized Variants as Full-Lengths IgGs

**Figure 7:**
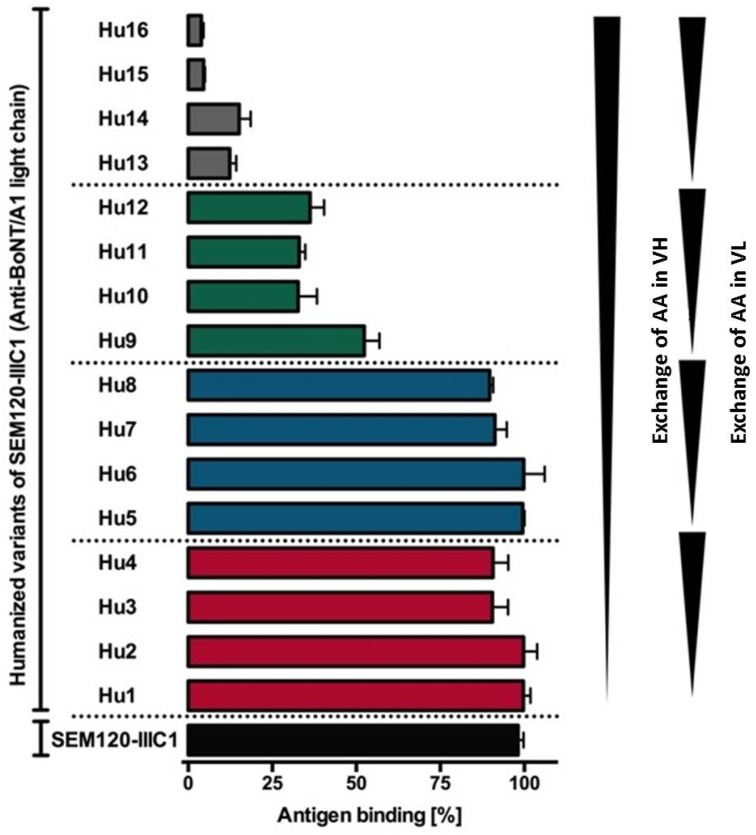
Antigen binding of humanized variants of SEM120-IIIC1 (anti-BoNT/A1 LC).

In a first step towards the humanization of the selected antibodies, we exchanged the AA in the FRs of SEM120-IIIC1, A1HC38, BLC3 and B2-7 with their human counterpart classified as very similar AA and similar AA. The resulting humanized variable domains were named hu1VH and hu1VL. We included the AA classified as dissimilar AA, resulting in the humanized variants hu2VH and hu2VL. In the case of SEM120-IIIC1, we decided to exchange the AA that was classified as very dissimilar AA, resulting in the humanized Variants hu3VH and hu3VL and modelled each variant using WAM antibody modelling [86]. In the next step, the humanized variable domains were all combined, including the parental VH and VL and produced as scFv-Fc. The resulting humanized variants were termed hu1SEM120-IIIC1 up to hu16SEM120 IIIC1. The antigen bindings of the 16 humanized variants were compared and validated by ELISA tests using immobilized recombinant LC or HC of BoNT and by surface plasmon resonance (SPR) analyses using holotoxin. Based on the ELISA and SPR analyses the hu8SEM120-IIIC1 was selected for further in vivo studies [87] (Figure 7).

Based on the experience drawn from the humanization of SEM120-IIIC1, we only used very similar, similar and dissimilar AA exchanges for the germline-humanization of A1HC38, BLC3 and B2-7. The resulting humanized antibody variants were named hu1A1HC38 up to hu8A1HC38, hu1BLC3 up to hu8BLC3 or hu1B2-7 up to hu8B2-7. The antigen binding of the eight humanized variants of A1HC38, BLC3 and B2-7 was compared and validated by ELISA using recombinant LC and HC of BoNT/A or B. Based on these results, the hu8A1HC38, hu8BLC3 and hu8B2-7 variants were selected for further in vivo studies. The average GI of 500 scFv, isolated out of the naïve human antibody gene library HAL7/8, was 96.8% (VH), 95.4% (VL lambda) and 94.8% (VL kappa). With GI values of 95% (hu8BLC3), 94.4% (hu8B2-7), 94.5% (hu8SEM120-IIIC1) and 94.9% (hu8AHC38) the germline-humanized variable domains are as human as naïve human germline derived variable domains.

**Figure 8:**
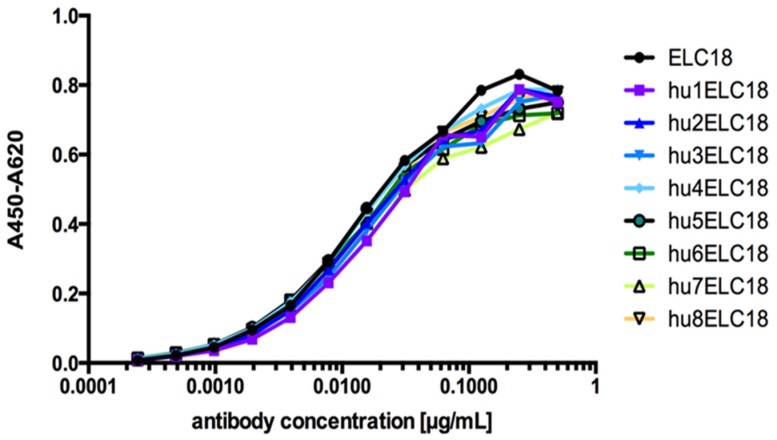
ELISA assay of all humanized ELC18 variants (from hu1ELC18 to hu8ELC18) and non-humanized ELC18. Modified figure from our previous publication: Derman et al. 2016 [76].

For the humanization process of ELC18 we used a multistep approach. In a first step, we designed humanized variants of the variable domains of ELC18 by exchanging AA in the FRs differ from the human germline sequence with their human counterpart classified as very similar and similar AA. The resulting variable domains were named hu1VH and hu1VL. We then included the AA classified as dissimilar AA, resulting in the humanized variants hu2VH and hu2VL, and combined each variable domain with each other, including the parental VH and VL. By exchanging this AA, the GI value of the humanized antibodies rose to 97.3% (hu8ELC18). To validate the quality of the humanization process, we produced eight distinct variants as scFv-Fc antibodies and the antigen binding of the eight humanized variants, and the parental ELC18 was compared and validated by ELISA using immobilized BoNT/E LC (Figure 8). No significant difference in the antigen binding was observed between the humanized variants and the parental ELC18 (no reactivity against BSA, data not shown). For hu8ELC18, only 5 AA of the parental antibody were retained and classified as very dissimilar AA. The average GI of 500 scFv isolated out of the naive human antibody gene library HAL7/8 was 96.8% (VH), 95.4% (VL lambda) and 94.8% (VL kappa). With the highest GI value of 97.3% of all ELC18 variants, hu8ELC18 was selected for further in vivo studies. Therefore, scFv-Fc hu8ELC18 was re-cloned and produced as human IgG to obtain a ‘better than human’ IgG [76].

### 4.3. Assessment of the Protection Induced by Germline-Humanized IgGs in Lethal and Non-Lethal In Vivo Assays, Individually and in Combination

The selected IgGs targeting BoNT/A LC (hu8SEM120-IIIC1) and HC (hu8A1HC38) were tested in vivo in the mouse flaccid paralysis protection model both individually and in combination, for synergistic effect against pure BoNT/A1 (0.4 LD50 per dose, 1.74 pg) toxin. The hu8A1HC38, selected IgG targeting the HC of BoNT/A1 toxin, provided complete protection at 100 μg, 50 μg and 10 μg per dose while only partial protection was observed at all lower doses tested (5 μg and 1 μg per dose). The hu8SEM120-IIIC1, selected IgG targeting the LC of BoNT/A1 did not fully protect against the paralysis induced by BoNT/A1 toxin, even at 100 μg of IgG per dose. Any dose of this antibody between 100 μg and 5 μg provided similar partial protection against BoNT/A1 induced paralysis with no benefit of dose increase. However, when these two IgGs were combined as mixture of hu8SEM120 IIIC1 and hu8A1HC38, full protection was observed at all doses used. The lowest dose ensuring full protection from in vivo paralysis was 0.1 μg of each IgG in combination (0.2 μg total IgG), whereas full protection was not observed at 1.0 μg of both antibody alone, confirming strong synergy between IgGs targeting the LC and the HC.

The selected IgGs targeting the BoNT/B LC (hu8BLC3) and HC (hu8B2-7) were also tested individually and in combination for synergistic effect against in vivo paralysis induced by complex BoNT/B2 (0.2 LD50 per dose). Complete protection was achieved with hu8BLC3 at 100 μg, 50 μg and 10 μg per dose and partial protection with two further 10-fold dilutions whereas hu8B2-7 antibody did not significantly protect mice at any of the concentrations used in this study from 100 μg to 0.1 μg per dose. However, the combination of hu8B2-7 and hu8BCL3 fully protected mice in vivo at 0.25 μg dose of each IgG (0.5 μg total IgG) where protection was not observed for each antibody alone at 1.0 μg dose.

To further investigate the in vivo neutralizing potency of the antibodies, the selected IgGs targeting BoNT/A LC (hu8SEM120-IIIC1) and HC (hu8A1HC38) were tested in vivo in mouse lethality assay both individually and in combination for a synergistic effect against complexed form of BoNT/A1 (0.4LD50, 1.4 pg per dose). Although partial protection was achieved with 25 μg and 2.5 μg of hu8SEM120-IIICI or 2.5 μg of hu8A1HC38, respectively, complete neutralization of BoNT/A1 lethal activity was only obtained by combining both antibodies. The lowest fully protective dose was 2.5 μg of each IgG (5 μg total IgG), whereas 25 μg of individual antibody only induced a partial protection or survival with strong symptoms of botulism intoxication (hu8A1HC38), thus confirming the synergistic effect between the two antibodies.

**Figure 9:**
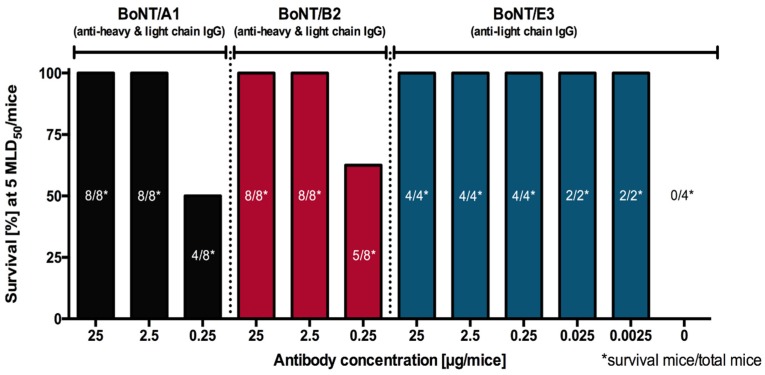
Survival rate of 5 MLD50/mice with a range of antibody concentrations.

The selected IgGs targeting the BoNT/B LC (hu8BLC3) and HC (hu8B2-7) were also tested individually and in combination for synergistic effect against lethality induced by complex BoNT/B2 (0.2 LD50 per dose). Doses of 25 μg and 2.5 μg of hu8BLC3 (anti-BoNT/B LC) provided partial protection against complex BoNT/B2 while extremely weak protection was observed with the same doses of hu8B2-7 (anti-BoNT/B HC) tested individually. However, the combination of hu8B2-7 and hu8BLC3 fully protected mice in vivo at 2.5 μg dose of each IgG (5 μg total IgG). Further reduced doses (0.25 μg of each IgG) still induced 50% protection against 5 MLD50 of BoNT/B2 (Figure 9).

The germline-humanized scFv-Fc ELC18 into a full IgG hu8ELC18 demonstrated protection and prophylaxis capacity against BoNT/E in a mouse model. A concentration of 2.5 ng/mouse of hu8ELC18 protected against 5 mouse lethal dose (MLD) in a mouse protection assay and complete neutralization of 1 LD50 of pure BoNT/E toxin was achieved with 8 ng of hu8ELC18 in mouse paralysis assay. Furthermore, hu8ELC18 protected mice from 5 MLD if injected up to 14 days prior to intraperitoneal BoNT/E administration [76] (Figure 9).

## 5. Output of the AntiBotABE Project

**Figure 10:**
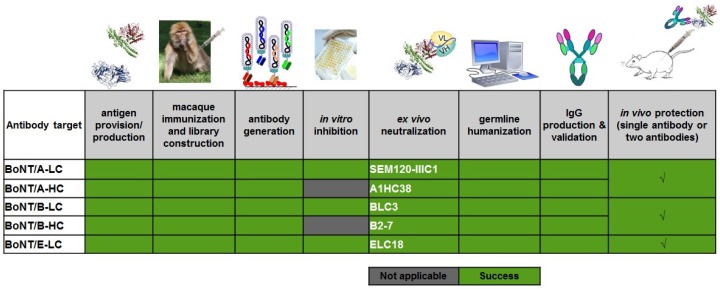
Overview of AntiBotABE output.

In the context of the AntiBotABE project, a previously successfully strategy was used to isolate neutralizing antibodies directed against the LC of BoNT/A, B and E and HC of BoNT/A and B (Figure 10).

Six immune phage-display libraries were constructed starting from macaques (*Macaca fascicularis*) immunized with a non-toxic recombinant fragment corresponding to BoNT/A1-HC, BoNT/A1-LC, BoNT/B2-HC, BoNT/B2-LC, BoNT/E3-HC and BoNT/E3-LC. The screening of immune libraries led to the isolation of several neutralizing scFvs except for the library directed against the heavy chain of BoNT/E3. Interestingly, antibodies that cross-neutralized both subtypes A1 and A2 of BoNT/A and B1 and B2 of BoNT/B were isolated. Such cross-neutralizing antibodies are highly sought-after in therapy to expand the therapeutic spectrum of each single antibody while decreasing the number of antibodies required to develop a wide spectrum neutralizing mixture. To ensure excellent bioavailability and tolerance the antibodies were germline-humanized then reformatted as full-sized IgG and successfully tested in vivo.

Protective antibody combinations against BoNT/A and BoNT/B were developed. For BoNT/E, the anti-LC antibody alone was highly protective. The combination of these five antibodies as oligoclonal antibody cocktail can be clinically developed for regulatory approval as a therapy.

Studies on recombinant antibodies neutralizing BoNT/B and E by targeting their light chains have not been published to date. Our studies demonstrated that scFv-Fc BLC3 at doses as low as 2.5 μg fully protected mice from paralysis induced by 0.2 LD50 of BoNT/B2 toxin. BLC3 demonstrated an affinity to the holotoxin BoNT/B1 that was over 100-fold higher than to the non-toxic immunogen, BoNT/B2-LC, which also suggests a preference to conformational epitopes on the whole toxin, and in turn may also contribute to its higher potency in vivo. High affinity of short chain antibody fragments is a key element of in vivo efficacy as previously reported for antibodies against BoNT/A-HC and LC [88].

An interesting observation was made regarding the anti-BoNT/B2 antibodies since we initially expected that the main protective effect against BoNTs would be based on the binding of the HC, which is responsible for receptor binding and translocation of the LC into the cytoplasm. The protective effect of the anti-LC antibody hu8BLC3 could be caused by binding an epitope close to the HC that interacts with receptor binding or inhibits the translocation of the LC into the cytoplasm. It is also possible that hu8BLC3 would be still blocking the endopeptidase activity of the LC in the cytoplasm.

To our knowledge, no recombinant human-like antibody neutralizing BoNT/E by targeting the LC (BoNT/E-LC) has been reported to date. Only neutralizing antibodies targeting the HC of BoNT/E were described, such as 4E17 isolated from a human immune library targeting a conserved epitope of the BoNT translocation domain [63], or BMR2, a single domain VHH from dromedary recognizing the binding domain of BoNT/E [89]. The anti-BoNT/E IgG hu8ELC18 showed high level of neutralization capacity of 5 MLD/mouse BoNT/E at concentrations as low as 0.0025 μg IgG/mouse. We also observed that hu8ELC18 showed protection when administered up to 14 days prior to BoNT/E challenge which suggests preventive effects [76]. This newly developed germline humanized IgG hu8ELC18 is predicted to have high tolerance in humans, can be produced in high quantities without the use of animals, and may therefore serve as a better therapeutic option to treat human botulism compared to HBAT [90].

The protection observed with the five antibodies isolated is outstanding, because it was achieved without affinity-improvement engineering. As a comparison, the murine antibody C25 directed against BoNT/A1-HC was humanized (HuC25) to enable therapeutic use and engineered to enhance its cross-reactivity with BoNT/A2. A mutant variant (CR2) was isolated with improved affinity to BoNT/A2 (from 109 nM to 87 pM), whilst retaining its affinity to BoNT/A1 (115 pM). It was later shown that the CR2 had the same inhibitory capacity against the enzymatic activity of BoNT/A1 and BoNT/A2 [52].

The demonstration of the protective synergistic effect during the AntiBotABE project is in good agreement with previous studies with an oligoclonal recombinant antibody preparation composed of 3 mAbs directed against BoNT/A [53] and studies on the combination of two mAbs directed against HC and the -LC domains of BoNT/A [54]. A combination of the four germline-humanized anti-BoNT/A and B IgGs with a formerly described anti-BoNT/E antibody would result in a promising oligoclonal antibody product that could be effective against the three main BoNTs serotypes involved in human botulism cases.

In combination, the germline-humanized IgG targeting the HC and LC domain of BoNT/A and B are neutralizing in vivo and it is expected that these IgGs are well tolerated in humans with less or no adverse effect. In contrast to usual therapeutics, such as BabyBIG^®^ or HBAT, they can be produced in large amount without the use of animals and are suitable for further clinical development as part of an oligoclonal drug for treatment of botulism. Such a drug would be helpful both for the European Union biodefense and for the treatment of natural botulism.

## 6. Dissemination of the AntiBotABE Project

The project has the potential to offer an unequalled level of security against bio-threats in Europe, based upon a family of well-tolerated and effective molecules. The synergistic effects and human-like nature of the selected IgGs makes them promising lead candidates for further clinical development.

- The first aim was to promote knowledge of our results among the potentials users of our antibodies, the governmental structures involved in bioterrorism preparedness, and the general public.
- In a next step, we addressed potential stakeholders for the further clinical and regulatory development of the AntiBotABE antibody cocktail (oligoclonal antibody). In order to draw the attention of European institutions, national governments, regional authorities and other public and private funding sources to the needs and benefits of our antibodies; and the need to stockpile them in advance: several presentations to scientific and decision-makers fora such as the European Defense Agency (EDA) and the North Atlantic Treaty Organization (NATO) have been made.
- Enhancing the reputation of participants at local, national and international levels and attracting the interest of correspondents, including the public; Aid the search for financial backers, licensees or industrial implementers to exploit the results and maintain market demand for the developed products or services. To this aim, regular up-dates of the AntiBotABE progress have been presented as oral presentations and posters at international meetings by the Consortium members.

## Figures and Tables

**Table 1:** Affinity, in vitro inhibition and ex vivo neutralization properties of selected scFvs or scFv-Fcs.

Antigen	Antibody	Format	In Vitro Inhibition: IC 50 (nM)	Ex Vivo Neutralization: Neutralizing Concentration 50% (nM)	Affinity (KD, nM)
BoNT/A LC	SEM120-IIIC1	scFv-Fc	10	1000	0.82
BoNT/A HC	AHC38	scFv	n/a	33	1.9
BoNT/B LC	BLC3	scFv-Fc	66	66	0.4
BoNT/B HC	B2-7	scFv	n/a	>1000	4.8
BoNT/E LC	ELC18	scFv	112	3.3	0.58
BoNT/E HC	-	n/a	n/a	n/a	n/a

BoNT/E HC: n/a due to lack of neutralization.

**Table 2:** Germinality indexes (GI %) of selected scFvs or scFv-Fcs.

Antigen	Antibody	Format	Germinality Index (GI %)
BoNT/A LC	SEM120-IIIC1	scFv-Fc	86.8 (VH)–87.6 (VL)
BoNT/A HC	AHC38	scFv	86.5 (VH)–84.4 (VL)
BoNT/B LC	BLC3	scFv-Fc	85.7 (VH)–85.7 (VL)
BoNT/B HC	B2-7	scFv	85.7 (VH)–77.2 (VL)
BoNT/E LC	ELC18	scFv	85.7 (VH)–89.9 (VL)
BoNT/E HC	-	n/a	n/a
